# Supplementary material for: Functional development of the human cerebellum from birth to age five
Source: Nat Commun. 2025 Jul 10;16:6350. doi: 10.1038/s41467-025-61465-y (PMC12246265; doi:10.1038/s41467-025-61465-y)
Supplement: Supplementary file 1 — Supplementary Information [file 41467_2025_61465_MOESM1_ESM.pdf]

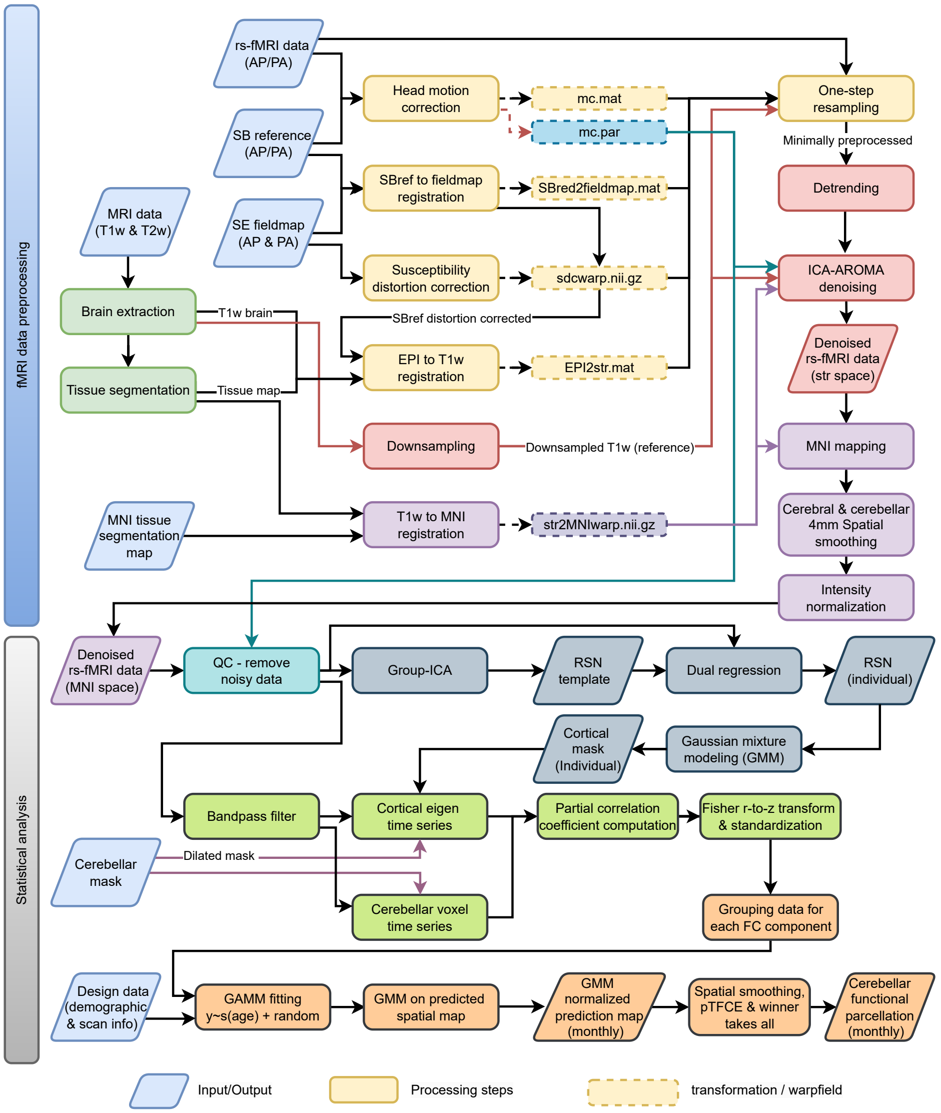

Fig. S1 | **Processing and analysis pipelines.** Steps involved in data processing and statistical analysis. T1w, T1-weighted; T2w, T2-weighted; rs-fMRI, resting-state functional magnetic resonance imaging; AP, anterior-posterior phase encoding; PA, posterior-anterior phase encoding; SB, single band; SE, spin echo; EPI, echo planar imaging; str, structural; ICA-AROMA, independent component analysis-based automatic removal of motion artifacts; QC, quality control; RSN, resting state network; GAMM, generalized additive mixed model; GMM, Gaussian mixture modeling; FC, functional connectivity; pTFCE, probabilistic threshold-free cluster enhancement.

Tab. S1 | Networks. Cortical networks and their corresponding primary regions.

Network	Abbreviation	Primary Regions
**Sensorimotor Network (SMN)**		
Foot	SM-Foot	Bilateral precentral and postcentral gyri
Left hand	SM-Hand-L	Left precentral and postcentral gyri
Right hand	SM-Hand-R	Right precentral and postcentral gyri
Tongue	SM-Tongue	Bilateral precentral and postcentral gyri
**Auditory Network (AUD)**		
Auditory	AUD	Bilateral superior temporal gyri
**Visual Network (VIS)**		
Medial occipital	VIS-Occ-Med	Bilateral cuneus and lingual gyri
Lateral	VIS-Lat	Bilateral cuneus, lingual gyri, and fusiform gyri
Superior occipital	VIS-Occ-Sup	Bilateral cuneus
Inferior occipital	VIS-Occ-Inf	Bilateral cuneus and lingual gyri
Occipital pole	VIS-Occ-Pol	Bilateral occipital poles
**Salience Network (SN)**		
Medial	SAL-Med	Anterior cingulate cortex and bilateral insulae
Lateral	SAL-Lat	Bilateral inferior frontal gyri and insulae
**Ventral Attention Network (VAN)**		
Frontal	VA-Front	Bilateral superior, middle, and inferior frontal gyri
Parietal	VA-Par	Bilateral paracentral lobes, supramarginal gyri, and insulae
**Default Mode Network (DMN)**		
Prefrontal	DM-Pref	Prefrontal cortex
Posterior cingulate	DM-Cing-Post	Posterior cingulate cortex and precuneus
Parietal angular	DM-Ang	Bilateral angular and middle temporal gyri
Left temporal	DM-Temp-L	Left middle temporal gyrus and temporal pole
Right temporal	DM-Temp-R	Right middle temporal gyrus and temporal pole
Parahippocampal	DM-PHippo	Bilateral parahippocampal, fusiform, and angular gyri
**Executive Control Network (ECN)**		
Prefrontal	EC-Pref	Prefrontal cortex
Left frontal	EC-Front-L	Left superior and middle frontal gyri
Right frontal	EC-Front-R	Right superior and middle frontal gyri
Supramarginal	EC-SMarg	Bilateral supramarginal gyri
Temporal	EC-Temp	Bilateral fusiform and inferior temporal gyri
**Dorsal Attention Network (DAN)**		
Medial parietal	DA-Par-Med	Bilateral precuneus and prefrontal cortices
Left parietal	DA-Par-L	Left superior parietal lobule
Right parietal	DA-Par-R	Right superior parietal lobule
Supramarginal	DA-SMarg	Bilateral supramarginal and inferior frontal gyri
Temporal	DA-Temp	Bilateral angular and inferior temporal gyri

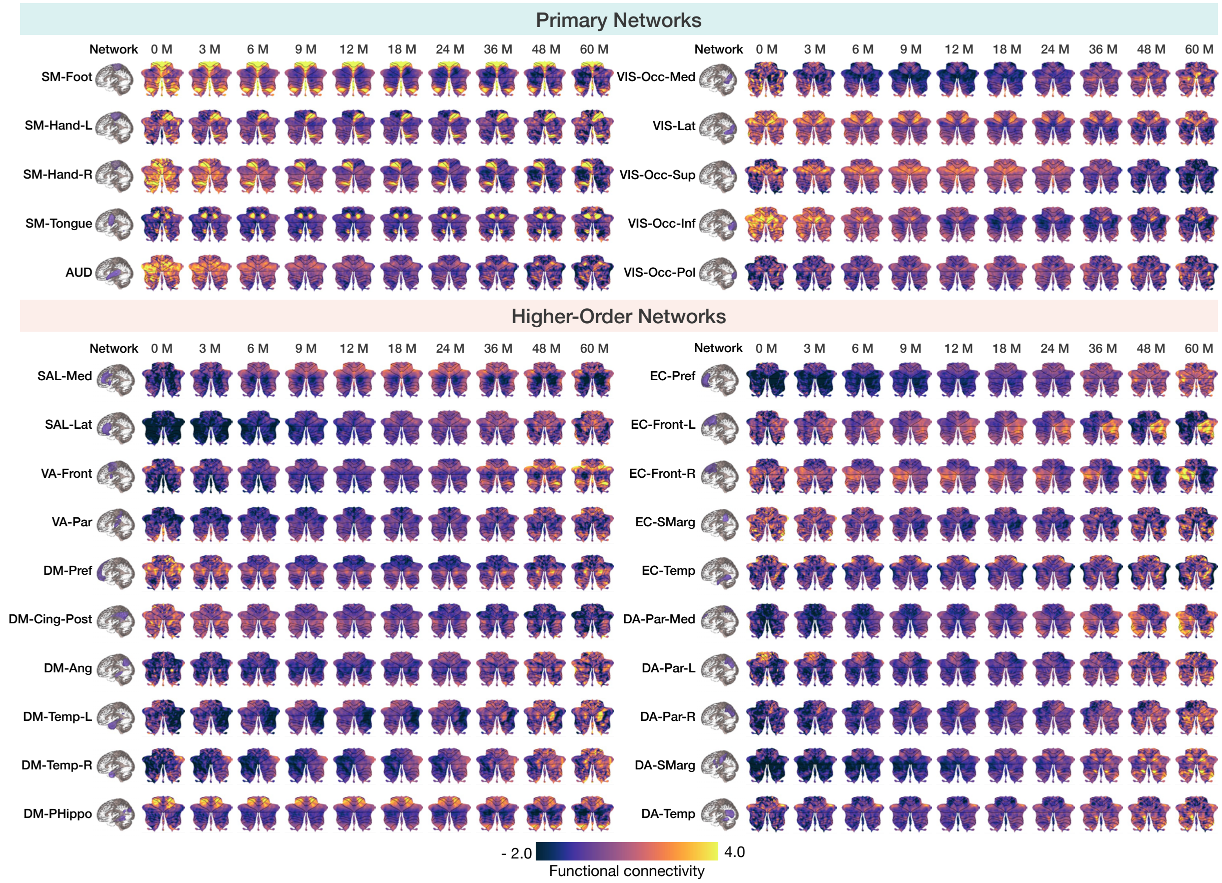

Fig. S2 | Cerebellar functional maps from birth to 60 months for children of both sexes. Spatiotemporal patterns of cerebellocortical functional connectivity (z-transformed) between the cerebellum and each RSN across early childhood. Values outside the range of -2.0 to 4.0 are capped for clarity.

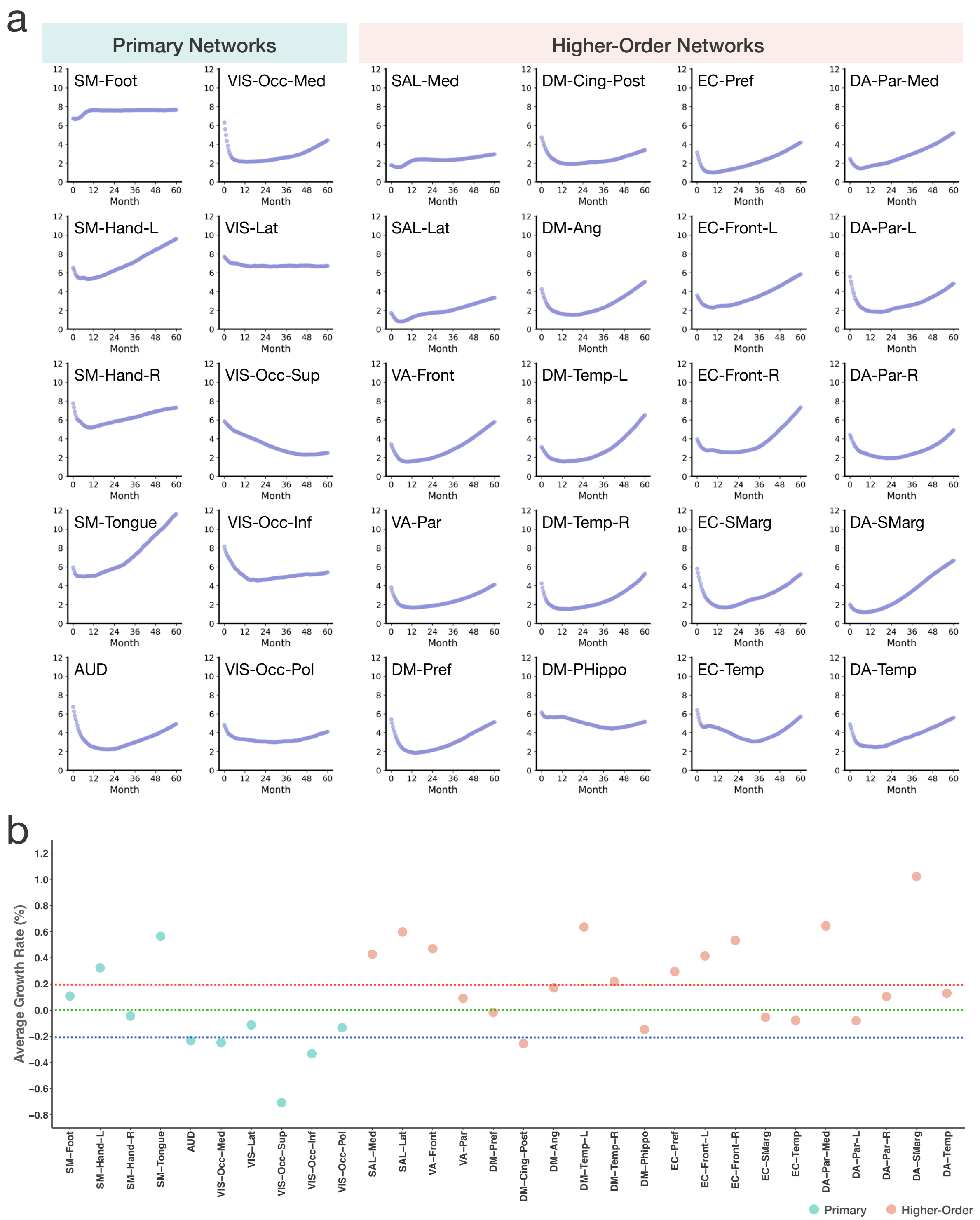

Fig. S3 | **Developmental trends of cerebellocortical functional connectivity.** **a**, Trajectories of the peak cerebellar connectivity (z-transformed) with each RSN over time. Each panel shows one RSN, with age in months on the horizontal axis and peak functional connectivity on the vertical axis. **b**, Average biweekly growth rate of cerebellar connectivity with each RSN. Values above the red dashed line denote substantial positive growth, values around the green dashed line denote negligible or minimal growth, and values below the blue dashed line denote substantial negative growth.

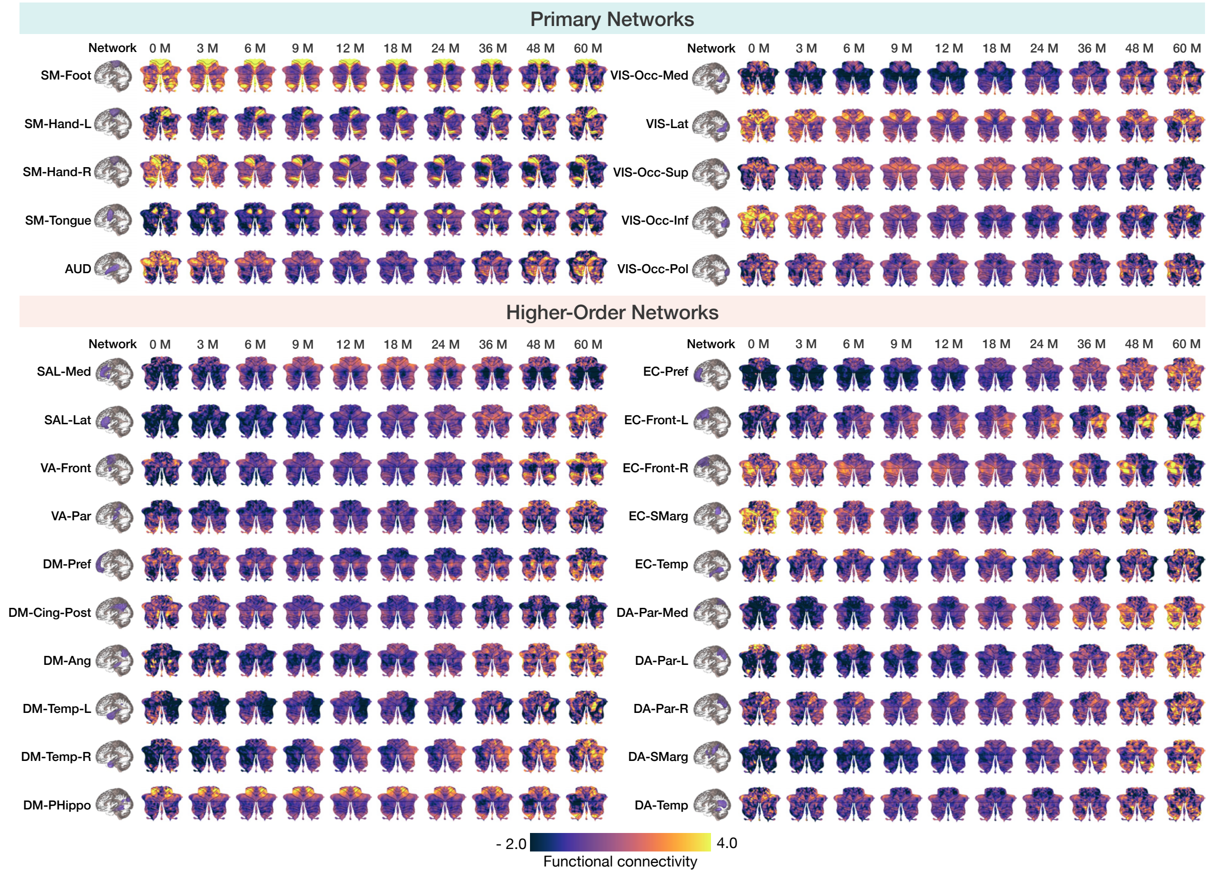

Fig. S4 | Cerebellar functional maps from birth to 60 months for female children. Spatiotemporal patterns of cerebellocortical functional connectivity (z-transformed) between the cerebellum and each RSN during early childhood in females. Values outside the range of −2.0 to 4.0 are capped for clarity.

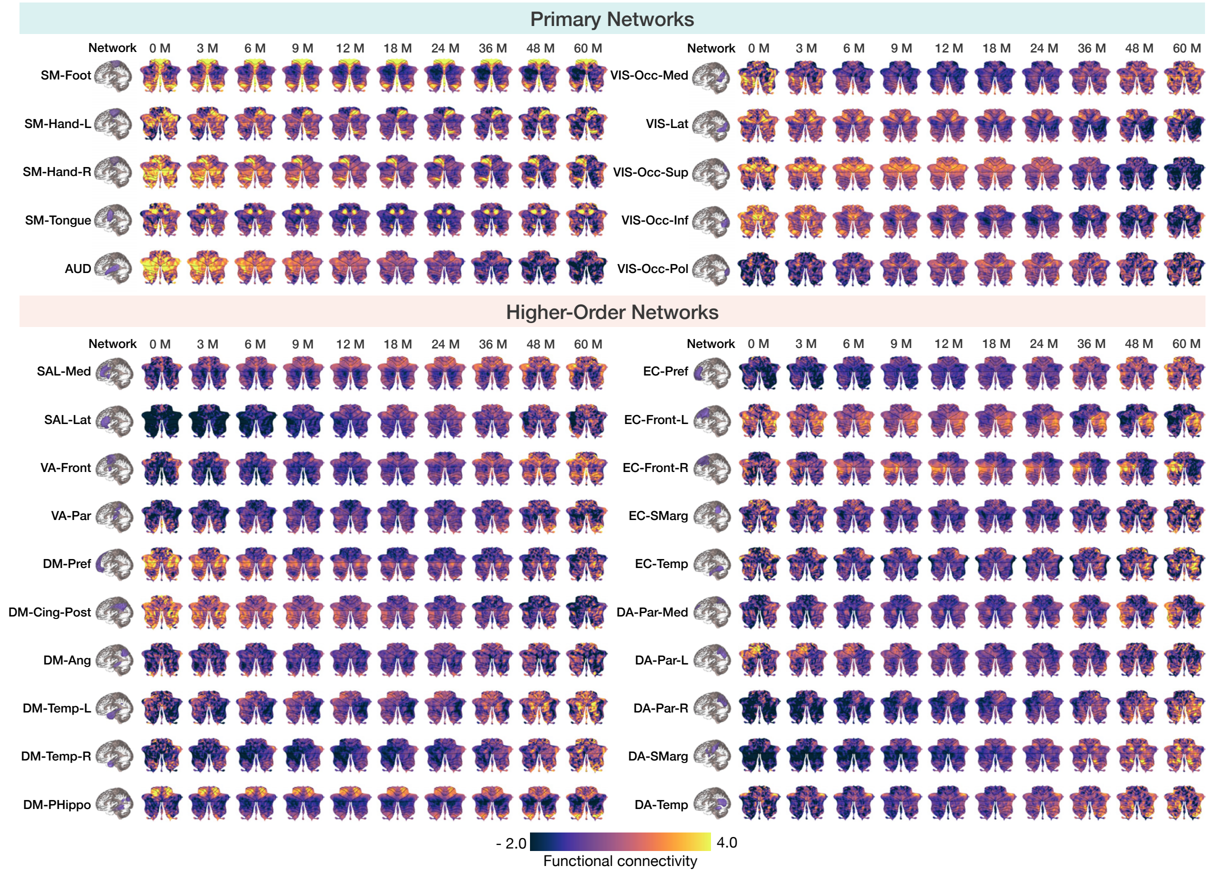

Fig. S5 | Cerebellar functional maps from birth to 60 months for male children. Spatiotemporal patterns of cerebellocortical functional connectivity (z-transformed) between the cerebellum and each RSN during early childhood in males. Values outside the range of -2.0 to 4.0 are capped for clarity.

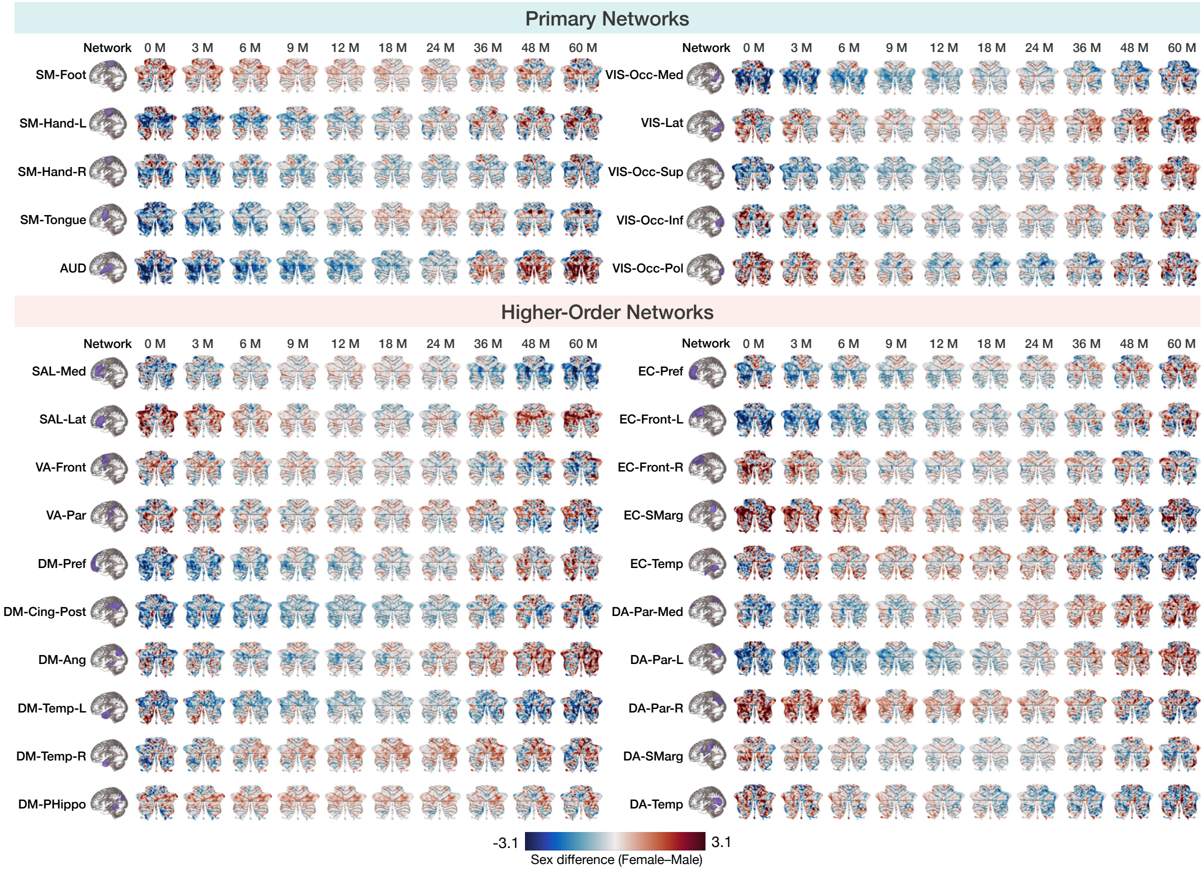

Fig. S6 | Sex differences spatial maps in cerebellocortical functional connectivity from birth to 60 months. Statistical significance was assessed using the one-tailed z-test under the assumption of a standard normal distribution. For clarity, values exceeding $\pm$ 3.1 (corresponding to $p < 0.001$) are capped. The color scale represents the direction and magnitude of the differences, with red indicating stronger connectivity in females and blue indicating stronger connectivity in males. Statistical significance was assessed using the one-tailed z-test under the assumption of a standard normal distribution.

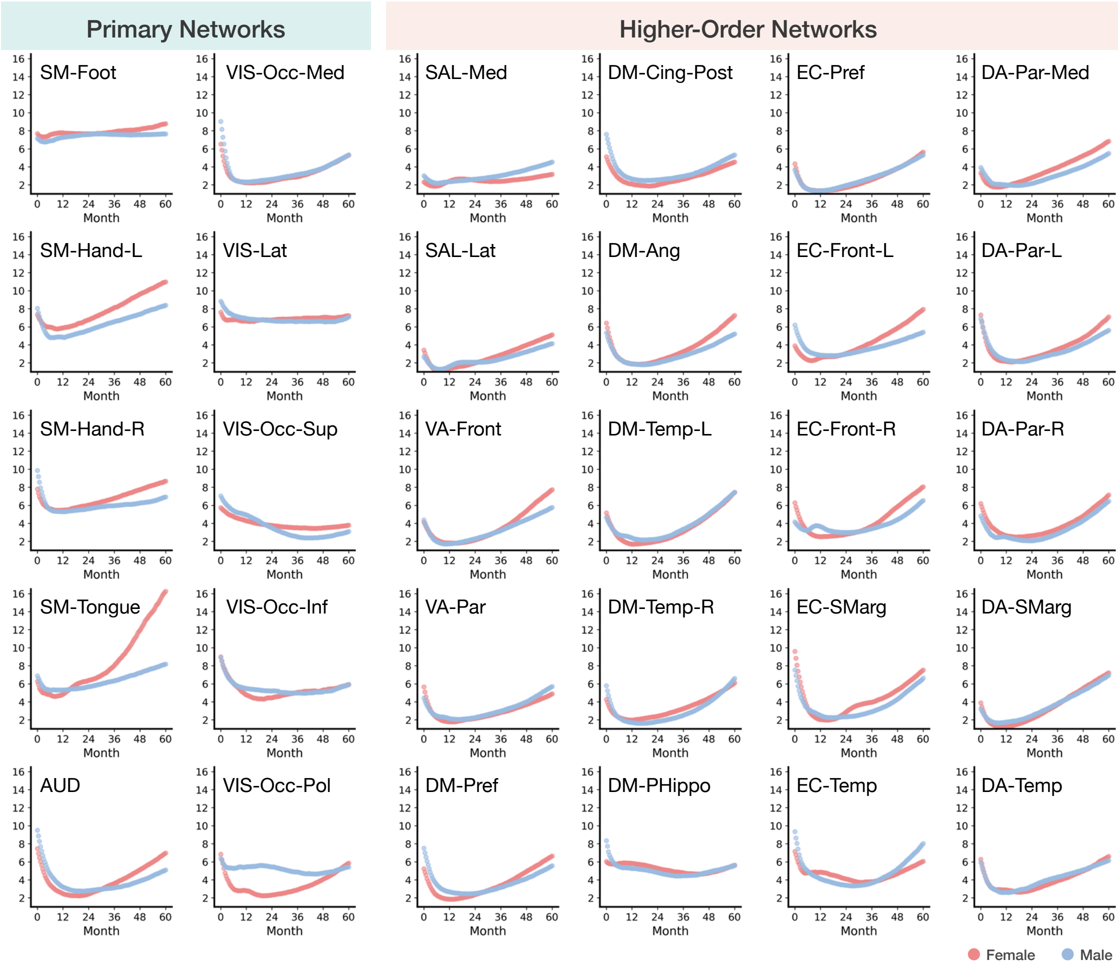

Fig. S7 | Developmental trends of cerebellocortical functional connectivity in female and male children. Trajectories of the peak cerebellar connectivity (z-transformed) with each RSN over time across female and male children. Each panel shows one RSN, with age in months on the horizontal axis and peak functional connectivity on the vertical axis.

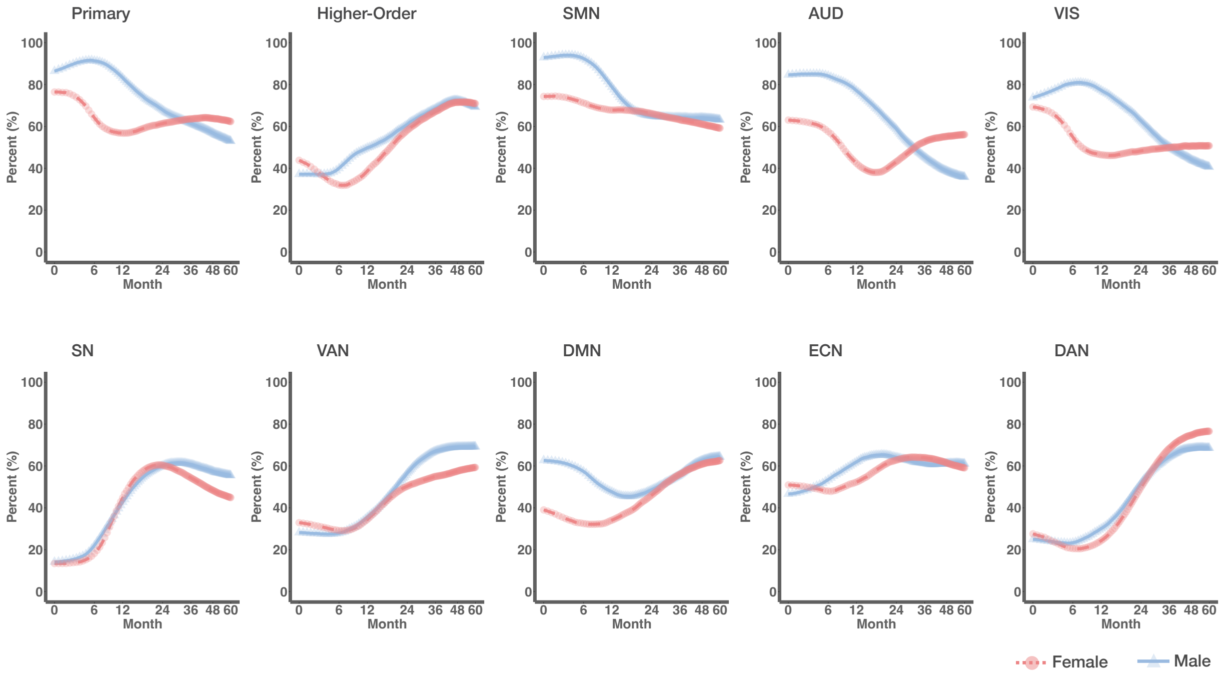

Fig. S8 | Sex-specific trajectories. Trajectories of cerebellar volume fractions of voxels with positive connectivity to cortical networks in female and male children.

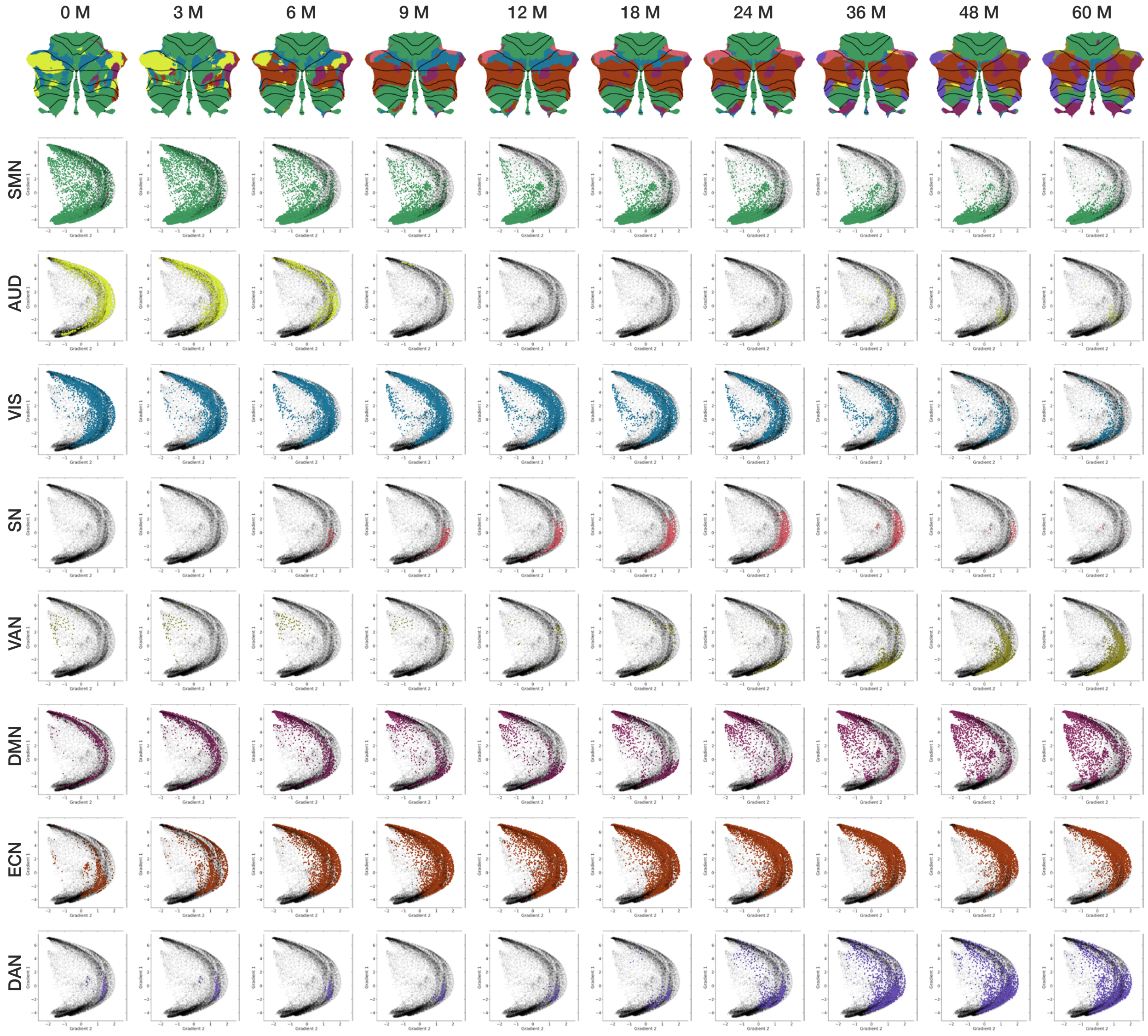

Fig. S9 | **Functional gradients.** Gradient maps of large-scale networks in children of both sexes over time.

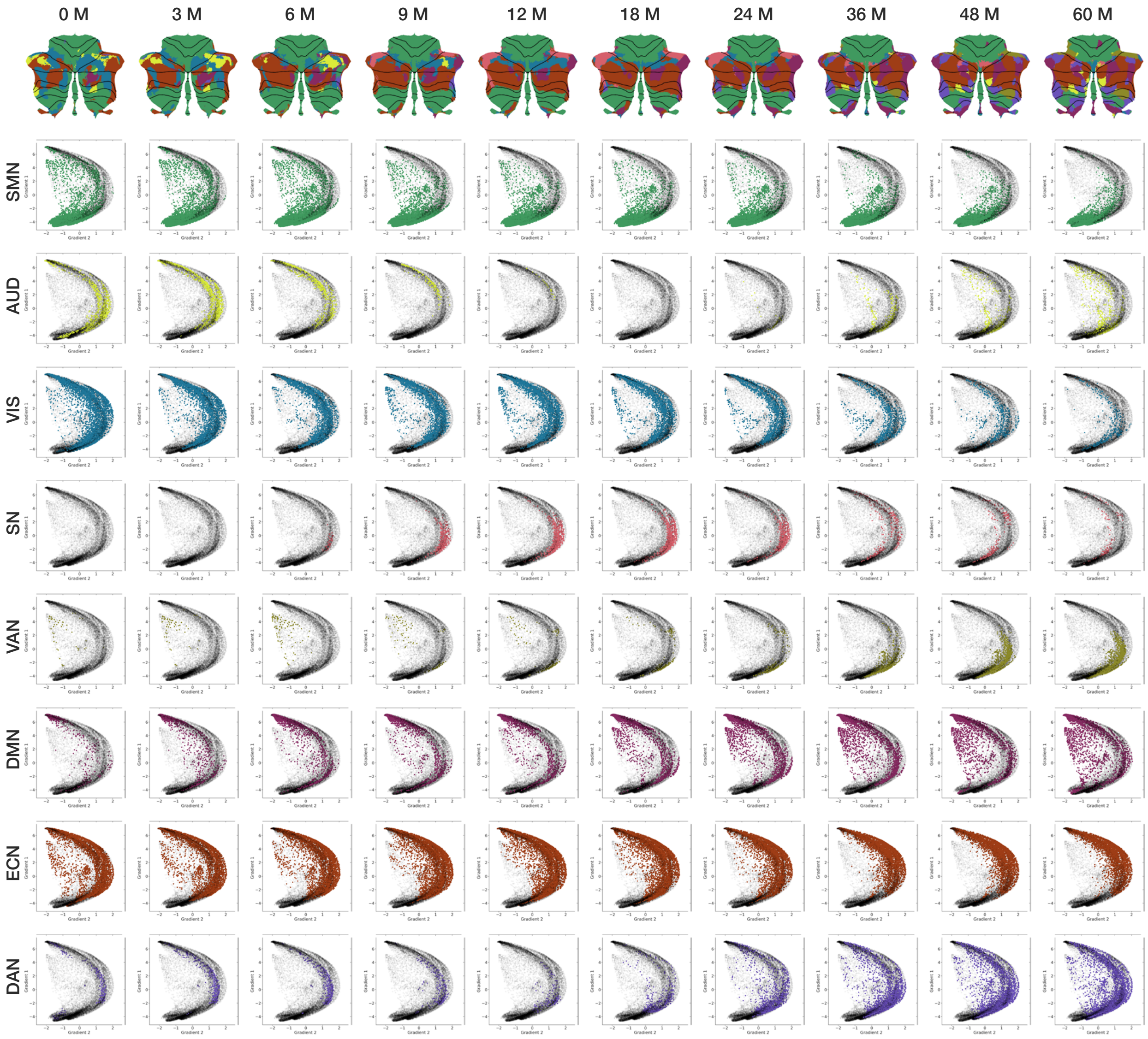

Fig. S10 | **Functional gradients.** Gradient maps of large-scale networks in female children over time.

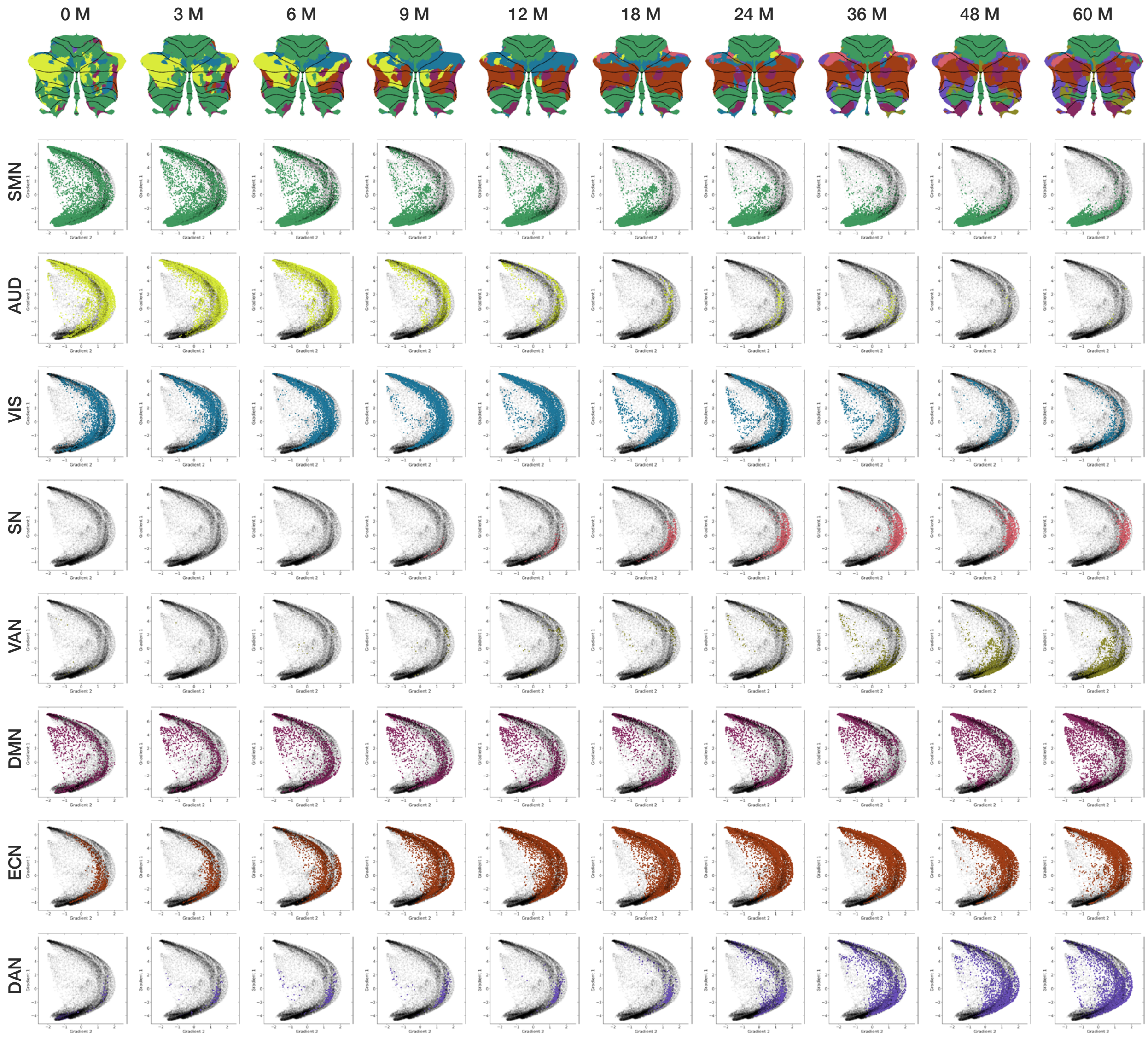

Fig. S11 | **Functional gradients.** Gradient maps of large-scales networks in male children over time.

Tab. S2 | Effects of wakefulness and motion. Percentage of cerebellar voxels showing significant associations (uncorrected $p < 0.01$) with wakefulness and motion. Associations were identified using two-tailed t-tests on regression coefficients from a voxel-wise linear model fit to GAMM residuals, with wakefulness and motion as covariates.

Component	Wakefulness	Motion
SM-Foot	0.00%	1.01%
SM-Tongue	0.00%	0.78%
SM-Hand-R	0.00%	1.03%
SM-Hand-L	0.00%	0.47%
AUD	0.00%	0.81%
VIS-Occ-Med	0.00%	0.47%
VIS-Lat	0.00%	2.31%
VIS-Occ-Inf	0.00%	1.17%
VIS-Occ-Pol	0.00%	0.71%
VIS-Occ-Sup	0.00%	0.67%
SAL-Med	0.00%	1.15%
SAL-Lat	0.00%	0.77%
VA-Front	0.00%	2.90%
VA-Par	0.00%	1.44%
DM-PHippo	0.00%	0.71%
DM-Ang	0.00%	0.63%
DM-Pref	0.00%	0.41%
DM-Cing-Post	0.00%	0.89%
DM-Temp-L	0.00%	0.76%
DM-Temp-R	0.00%	0.46%
EC-Pref	0.00%	0.69%
EC-Temp	0.00%	0.81%
EC-Front-R	0.00%	1.09%
EC-SMarg	0.00%	0.66%
EC-Front-L	0.00%	0.48%
DA-SMarg	0.00%	0.55%
DA-Temp	0.00%	3.67%
DA-Par-L	0.00%	0.57%
DA-Par-R	0.00%	0.74%
DA-Par-Med	0.00%	0.49%

1

Tab. S3 | **Correlation with Mullen scores.** Pearson correlations between peak cerebelloccortical connectivity and Mullen scores, adjusted for age using linear mixed models (bold if significant, uncorrected $p < 0.05$, two-tailed t-test). At coarse granularity, cerebellar connectivity with primary networks negatively correlates with fine motor scores, whereas connectivity with higher-order networks positively correlates with expressive language scores. At medium granularity, cerebellar connectivity with the SMN negatively correlates with fine motor scores, connectivity with the VAN negatively correlates with receptive language scores, and connectivity with the DAN positively correlates with both visual reception and expressive language scores.

Network	Gross Motor	Fine Motor	Visual Reception	Expressive Language	Receptive Language
Primary	-0.056/0.158	**-0.091/0.023**	-0.036/0.371	0.028/0.478	-0.054/0.174
Higher-Order	0.018/0.652	0.004/0.912	0.018/0.650	**0.105/0.008**	-0.055/0.169
SMN	-0.030/0.457	**-0.090/0.025**	-0.016/0.693	-0.026/0.522	-0.027/0.493
AUD	-0.029/0.473	-0.059/0.142	0.008/0.837	0.026/0.513	-0.039/0.331
VIS	-0.018/0.653	-0.054/0.177	-0.016/0.687	0.064/0.112	-0.027/0.506
SN	0.015/0.711	0.000/0.995	0.022/0.586	-0.044/0.274	-0.017/0.679
VAN	0.016/0.686	0.036/0.368	0.051/0.207	0.034/0.399	**-0.095/0.017**
DMN	0.005/0.907	-0.028/0.485	0.042/0.294	0.062/0.120	-0.068/0.090
ECN	0.025/0.525	-0.011/0.780	-0.015/0.711	0.022/0.588	-0.037/0.358
DAN	0.036/0.367	-0.006/0.888	**0.090/0.024**	**0.151/0.000**	-0.036/0.375
