# Supplementary material for: Functional development of the human cerebellum from birth to age five
Source: Nat Commun. 2025 Jul 10;16:6350. doi: 10.1038/s41467-025-61465-y (PMC12246265; doi:10.1038/s41467-025-61465-y)
Supplement: Supplementary file 2 — Reporting Summary [file 41467_2025_61465_MOESM2_ESM.pdf]

Corresponding author(s): Pew-Thian Yap

Last updated by author(s): May 31, 2025

Reporting Summary

Nature Portfolio wishes to improve the reproducibility of the work that we publish. This form provides structure for consistency and transparency in reporting. For further information on Nature Portfolio policies, see our Editorial Policies and the Editorial Policy Checklist.

Statistics

For all statistical analyses, confirm that the following items are present in the figure legend, table legend, main text, or Methods section.

n/a	Confirmed	
☐	☒	The exact sample size (*n*) for each experimental group/condition, given as a discrete number and unit of measurement
☐	☒	A statement on whether measurements were taken from distinct samples or whether the same sample was measured repeatedly
☐	☒	The statistical test(s) used AND whether they are one- or two-sided<br>*Only common tests should be described solely by name; describe more complex techniques in the Methods section.*
☐	☒	A description of all covariates tested
☐	☒	A description of any assumptions or corrections, such as tests of normality and adjustment for multiple comparisons
☐	☒	A full description of the statistical parameters including central tendency (e.g. means) or other basic estimates (e.g. regression coefficient) AND variation (e.g. standard deviation) or associated estimates of uncertainty (e.g. confidence intervals)
☐	☒	For null hypothesis testing, the test statistic (e.g. *F*, *t*, *r*) with confidence intervals, effect sizes, degrees of freedom and *P* value noted<br>*Give P values as exact values whenever suitable.*
☒	☐	For Bayesian analysis, information on the choice of priors and Markov chain Monte Carlo settings
☒	☐	For hierarchical and complex designs, identification of the appropriate level for tests and full reporting of outcomes
☐	☒	Estimates of effect sizes (e.g. Cohen's *d*, Pearson's *r*), indicating how they were calculated

Our web collection on statistics for biologists contains articles on many of the points above.

Software and code

Policy information about availability of computer code

Data collection	No software was used
Data analysis	FSL(6.0), ICA-AROMA (https://github.com/maartenmennes/ICA-AROMA), ANTs (2.2.0, https://github.com/ANTsX/ANTs), Matlab (2023a; toolboxes: SPM12(https://www.fil.ion.ucl.ac.uk/spm/software/spm12/), SUIT(https://github.com/jdiedrichsen/suit)), LittleBrain (https://github.com/xaviergp/littlebrain), Python(3.12), R (4.4.0; packages: gamm4, mgcv, bigmemory, ggplot2, fslr, pTFCE, oro.nifti, R.matlab, matlib, dplyr, and pryr.), and MRIcroGL(1.2,https://www.nitrc.org/projects/mricrogl/).

For manuscripts utilizing custom algorithms or software that are central to the research but not yet described in published literature, software must be made available to editors and reviewers. We strongly encourage code deposition in a community repository (e.g. GitHub). See the Nature Portfolio guidelines for submitting code & software for further information.

Data

Policy information about availability of data

All manuscripts must include a data availability statement. This statement should provide the following information, where applicable:

- Accession codes, unique identifiers, or web links for publicly available datasets
- A description of any restrictions on data availability
- For clinical datasets or third party data, please ensure that the statement adheres to our policy

The BCP dataset used in this study is available from the National Institute of Mental Health Data Archive (NDA, https://nda.nih.gov). Processed data supporting the findings of this study is available at Figshare (https://doi.org/10.6084/m9.figshare.25970518). Source data are provided with this paper.

Research involving human participants, their data, or biological material

Policy information about studies with human participants or human data. See also policy information about sex, gender (identity/presentation), and sexual orientation and race, ethnicity and racism.

Reporting on sex and gender	Sex was considered in the study design. Sex-specific developmental patterns were explicitly examined both in the study design and in the data analysis.
Reporting on race, ethnicity, or other socially relevant groupings	Race, ethnicity, or any other socially relevant groupings were not included in this study.
Population characteristics	275 subjects from birth to 5 years old (130 males, 145 females) enrolled as part of the Baby Connectome Project (BCP).
Recruitment	Participants in the BCP were recruited from existing registries at UNC and UMN based on state-wide birth records as well as from broader community resources (e.g., community centers and targeted day-care centers) to ensure the sample approximates the racial/ethnic and socio-economic diversity of the US census.
Ethics oversight	Ethical approval for all study procedures was granted by the institutional review boards of the University of North Carolina at Chapel Hill (UNC) and the University of Minnesota (UMN).

Note that full information on the approval of the study protocol must also be provided in the manuscript.

Field-specific reporting

Please select the one below that is the best fit for your research. If you are not sure, read the appropriate sections before making your selection.

☒ Life sciences ☐ Behavioural & social sciences ☐ Ecological, evolutionary & environmental sciences

For a reference copy of the document with all sections, see nature.com/documents/nr-reporting-summary-flat.pdf

Life sciences study design

All studies must disclose on these points even when the disclosure is negative.

Sample size	We collected data from a well-characterized cohort of children aged from birth to 5 years. No formal statistical method was used to predetermine sample size. Instead, the sample size was determined by the availability of high-quality data passing rigorous preprocessing and quality control. The final dataset includes 1,017 functional MRI scans, which constitutes a relatively large sample size appropriate for group-level analyses.
Data exclusions	Subjects with fMRI data showing excessive motion, i.e., mean Power's FD (absolute sum of motion parameters) > 0.5mm and mean Jenkinson's FD > 0.2mm, were excluded. Samples not passing visual inspection due to excessive image distortion or failed registration were removed. Samples with NaN partial correlations were removed.
Replication	This is an observational cohort study based on a large-scale resting-state fMRI dataset from the Baby Connectome Project. As the study is observational and uses existing data, experiments were not replicated in the traditional sense. Instead, reproducibility was supported through rigorous preprocessing, quality control, and statistical modeling using generalized additive mixed models (GAMMs). To further validate the reliability of our analytic framework, we localized cerebellar sensorimotor representations (foot, hand, tongue) and confirmed that their topography matched well-established adult patterns [Yeo et al., 2011; Xue et al., 2021].
Randomization	We did not divide our dataset into multiple groups. No randomization was involved
Blinding	Our study involved only healthy individuals with a constant experimental condition (resting-state fMRI). Thus, no blinding was necessary.

Reporting for specific materials, systems and methods

We require information from authors about some types of materials, experimental systems and methods used in many studies. Here, indicate whether each material, system or method listed is relevant to your study. If you are not sure if a list item applies to your research, read the appropriate section before selecting a response.

Materials & experimental systems

n/a	Involved in the study
x	Antibodies
x	Eukaryotic cell lines
x	Palaeontology and archaeology
x	Animals and other organisms
x	Clinical data
x	Dual use research of concern
x	Plants

Methods

n/a	Involved in the study
x	ChIP-seq
x	Flow cytometry
	x MRI-based neuroimaging

Plants

Seed stocks: N/A

Novel plant genotypes: N/A

Authentication: N/A

Magnetic resonance imaging

Experimental design

Design type: resting-state

Design specifications: Each resting-state fMRI scan was acquired in 5min 47s, and consisted of 420 volumes. Children younger than 3 years of age were imaged during natural sleep while older subjects (> 3 years old) were imaged during passive movie watching during rs-fMRI acquisition. Different phase-encoding directions (anterior-to-posterior (AP), and posterior-to-anterior (PA)) were used, generating at least two rs-fMRI data sets in each visit. For each phase-encoding direction, a single-band reference image (SBref) with identical voxel resolution, image dimension, phase encoding polarity and echo spacing with the rs-fMRI data was also acquired for motion correction. In addition, a pair of spin echo field maps with reversed phase-encode blip (i.e., AP and PA) were also acquired for distortion correction of rs-fMRI data caused by gradient nonlinearity and B0 inhomogeneity. Resting-state scan sessions (AP/PA) might be repeated for 2-3 times whenever possible, e.g., if the subject remains still.

Behavioral performance measures: No task during scanning.

Acquisition

Imaging type(s): Functional and structural

Field strength: 3T

Sequence & imaging parameters: T1w images were scanned with isotropic resolution of 0.8 mm, 320x320 matrix, 256 mm x 256mm FOV, 2.24ms TE, and 2400/1060 ms TR. T2w images were scanned with isotropic resolution of 0.8 mm, 320 x 320 matrix, 256mm x 256 mm FOV, 564 ms TE, and 3200 ms TR. Rs-fMRI data were collected using a single-shot echo-planar imaging (EPI) sequence, with isotropic resolution of 2 mm, 104 x 104 matrix, 208 mm x 208 mm FOV, 37 ms TE, 800 ms TR, 52 degree flip angle, multiband acceleration factor 8, acquisition time 5 min 47s, and 420 volumes.

Area of acquisition: whole-brain scans were acquired.

Diffusion MRI: ☐ Used ☒ Not used

Preprocessing

Preprocessing software: For structural MRI data, deep learning was used for tissue segmentation. For rs-fMRI data, FSL was used for preprocessing, ANTs was used to register structural data to MNI space, and ICA-AROMA was used for denoising.

Normalization	The denoised fMRI data was mapped into the MNI space, spatially smoothed for cerebal and cerebellar regions separately with FWHM of 4mm, and underwent intensity normalization for a constant mean volume intensity value of 10,000.
Normalization template	MNI152 (or ICBM 2009c nonlinear symmetrical).
Noise and artifact removal	FSL mcflirt for motion correction, FSL topup for EPI distortion correction, and ICA-AROMA for artifact removal.
Volume censoring	No volume censoring.

Statistical modeling & inference

Model type and settings	Generative additive mixture modeling (GAMM) fitting using partial correlation coefficients as response, and age, scan site, and subject ID as covariates. Age was the fixed effect. Scan site and subject ID were random effects. GAMM predicted spatial maps were normalized using weighted null mean and variance obtained from Gaussian Mixture Modeling. For parcellation, we first applied spatial smoothing at FWHM of 7 mm, and then performed pTFCE to obtain enhanced p-value maps.
Effect(s) tested	N/A.

Specify type of analysis: ☐ Whole brain ☐ ROI-based ☒ Both

Anatomical location(s): Determined based on the MNI space.

Statistic type for inference (See Eklund et al. 2016)	t-statistics
Correction	N/A

Models & analysis

n/a	Involved in the study
☐	☒ Functional and/or effective connectivity
☒	☐ Graph analysis
☒	☐ Multivariate modeling or predictive analysis

Functional and/or effective connectivity	We used partial Pearson's correlation coefficient to measure functional connectivity.
